# Risk Factors of Pre-eclampsia: A Hospital-Based Case-Control Study

**DOI:** 10.7759/cureus.42543

**Published:** 2023-07-27

**Authors:** Satish C Padhan, Pranati Pradhan, Bharati Panda, Subrat K Pradhan, Sanjeeb K Mishra

**Affiliations:** 1 Community Medicine, Veer Surendra Sai Institute of Medical Sciences and Research, Sambalpur, IND; 2 Obstetrics and Gynaecology, Veer Surendra Sai Institute of Medical Sciences and Research, Sambalpur, IND; 3 Field Epidemiology Training Program, Indian Council of Medical Research, Chennai, IND

**Keywords:** case-control studies, maternal morbidity, maternal death, maternal health, risk factor, pre-eclampsia

## Abstract

Introduction

Pre-eclampsia is a pregnancy-specific hypertensive disorder and is one of the leading causes of maternal and infant morbidity and mortality in India and worldwide. Evidence of the association between various risk factors and pre-eclampsia is scarce in developing countries. As pre-eclampsia remains a leading cause of maternal mortality and morbidity, focusing on the causes and risk factors of pre-eclampsia during antenatal surveillance would prevent maternal deaths and reduce the maternal mortality rate. Our study aimed to determine the risk factors of pre-eclampsia.

Materials and methods

An unmatched case-control study was conducted at Veer Surendra Sai Institute of Medical Sciences and Research (VIMSAR), Burla, Odisha, taking 100 cases of pre-eclampsia and 100 controls without pre-eclampsia from January 2021 to January 2023. The study population included patients admitted to the Obstetrics & Gynecology labor room. Study participants were selected randomly from the labor room thrice weekly. Data were collected using a predesigned pre-tested questionnaire and case report format. Data were analyzed using IBM SPSS Statistics for Windows, Version 26 (Released 2019; IBM Corp., Armonk, New York, United States). Appropriate statistical tests (Odds ratio, proportions, Chi-square test) were applied, and the final interpretation was made.

Results

Family history of hypertension (AOR = 4.2), history of chronic hypertension (AOR = 13.7), and AB blood group (AOR = 3.6) were found to be significant risk factors for pre-eclampsia. No significant association was found between pre-eclampsia and factors such as mother's age, caste, mother's education, type of family, socioeconomic status, education and occupation of husband, family history of diabetes mellitus, parity, history of abortion, and anemia.

Conclusion

Risk factors identified in the present study can be used to identify women at risk of pre-eclampsia during antenatal check-ups to minimize the complications of pre-eclampsia in both the mother and the fetus.

## Introduction

Pre-eclampsia is a pregnancy-specific hypertensive disorder usually occurring after 20 weeks of gestation. It is a multisystem disorder of unknown etiology characterized by the development of hypertension up to 140/90mm Hg or more with proteinuria after the 20 week of gestation in a previously normotensive and non-proteinuric pregnant woman. Pre-eclampsia has been associated with intrauterine growth retardation, preterm birth, and maternal and perinatal death [1]. India has a high maternal mortality ratio (MMR) of 97 per 1 lakh live births, and that of Odisha is 119 as per sample registration system (SRS) 2018- 20 [2]. As per sustainable development goals (SDGs), the target is to reduce the global MMR to less than 70 per 1 lakh live births by 2030 [3]. More focus should be given to the causes of maternal mortality to achieve this target, and necessary steps should be taken to prevent maternal deaths. Hemorrhage, sepsis, and hypertensive disorder of pregnancy are the leading causes of maternal mortality. Among the hypertensive disorders, pre-eclampsia and eclampsia have the most significant impact on maternal and newborn morbidity and mortality [4]. Pre-eclampsia, if not detected early, can lead to eclampsia which is one of the severe and direct causes leading to maternal and infant mortality and morbidity. About 5 to 10 percent of all pregnancies are complicated by pre-eclampsia and other hypertensive disorders [5].

Pre-eclampsia can lead to complications like antepartum and postpartum hemorrhage, eclamptic seizures, hepatic failure, acute renal failure, stroke, placental abruption, HELLP syndrome, heart failure, multiorgan failures, and maternal death. If not treated early, it can also cause fetal complications like fetal distress, intrauterine growth retardation, neonatal asphyxia, preterm birth, stillbirth, and perinatal death. Pre-eclampsia remains a substantial public health problem in both developing and developed countries. The incidence of pre-eclampsia in developing countries is seven times higher than that in developed countries, as per WHO estimates [6]. The impact of pre-eclampsia, i.e., maternal mortality, morbidity, and fetal complications, is more severely felt in developing countries. This might be due to the late detection of cases and ineffective treatment. Though it is tough to prevent pre-eclampsia, early detection and proper treatment can prevent its severity and complications. Hence with timely and effective care majority of the deaths related to pre-eclampsia and eclampsia can be avoided.

Various factors have been postulated to influence the risk factors of pre-eclampsia in various studies. These include the age of the mother being greater than 30 years, family history of hypertension, family history of diabetes mellitus, personal history of hypertension, history of hypertension in a previous pregnancy, primiparity, anemia during pregnancy, obesity, low physical activity, and maternal diet [7-10]. However, evidence of the association between the above risk factors and pre-eclampsia is scarce in developing countries. The knowledge and identification of various risk factors can help estimate each woman's individualized risk, allow antenatal surveillance to be directed at these women, and guide the healthcare providers to counsel such women and possibly reduce the risk of pre-eclampsia and its complications. As pre-eclampsia remains a crucial problem in maternal mortality and morbidity, giving attention to the causes and risk factors of pre-eclampsia during antenatal surveillance could expedite the reduction of the MMR. Since the causes and risk factors for pre-eclampsia remain unclear and there is an inadequacy of data on risk factors of pre-eclampsia in India and Odisha, this study was conducted to determine the risk factors of pre-eclampsia.

## Materials and methods

We conducted this unmatched case-control study in the labor room, VIMSAR, Burla, Sambalpur, Odisha, India. The study was conducted from January 2021 to January 2023. The cases included pregnant women diagnosed with pre-eclampsia after the 20th week of gestation, i.e., blood pressure ≥ 140/90 mm Hg on two occasions at least four hours apart and urinary protein ≥ 300mg in 24hr period or ≥ 1+ on a urine dipstick. Controls included all pregnant women admitted to the labor room after the 20th week of gestation and not diagnosed with pre-eclampsia. Women who were critically ill and cognitively impaired and had a history of cardiovascular diseases, endocrine diseases, autoimmune diseases, renal diseases, AIDS, and cancer were excluded from the study. Cases for data collection were sourced thrice weekly from the labor room of the O&G Department. On each day of data collection, a maximum of two pre-eclampsia patients satisfying inclusion criteria were selected randomly from the register in the labor room and were enrolled as study subjects. Recordings of the identifier data (name, age, address, registration details), disease details, and previous treatment were collected. Controls were selected from the register in the O&G labor room thrice weekly. A maximum of two controls were enrolled on each day of data collection. Controls were selected randomly from the register in the labor room. EPI INFO 7 was used to calculate the sample size by assuming primigravida as a risk factor with the lowest odds ratio of 2.5 and 39% exposure among controls from the reviewed literature [11]. With 95% confidence interval assumptions, 5% marginal error, 80% power, and 10% non-response final sample size of 188 were estimated, rounding up to 100 cases and 100 controls.

Data sources and data collection method

The structured interview questionnaire included sociodemographic factors, medical history, and gestational factors to collect data from each study participant, and the same questionnaire extracted data from patients' documents and indoor case sheets. Information on sociodemographic, behavioral, and clinical characteristics was collected through interviews from patient's documents and MCP cards. Blood pressure was measured using a sphygmomanometer. A urine dipstick was used to calculate urinary protein. Additional data on the patient's clinical condition were abstracted from the case sheet in the labor room. At the end of each interview, the principal investigator crosschecked the questionnaire to ensure completeness and data accuracy.

Data analysis

IBM SPSS Statistics for Windows, Version 26 (Released 2019; IBM Corp., Armonk, New York, United States) was used for data analysis after data cleaning. A descriptive analysis included frequency distribution, mean and standard deviation. The crude odds ratio (OR), Chi-square test for categorical variables (nominal data) at 95% confidence interval (CI), and alpha level of significance set at 0.05 were used as measures of association in the analysis of factors associated with pre-eclampsia. Univariate logistic regression determined the crude OR for those factors with p-value < 0.1 in the chi-square test. The final analysis was done using multivariable logistic regression for those factors with p-value < 0.1 in univariate logistic regression and using enter method to arrive at the factors that were independent predictors of pre-eclampsia. The final model contained only statistically significant factors after adjusting for confounding.

## Results

The study included 100 pre-eclampsia cases and 100 controls. The mean age in cases was 27.15 ± 5.15 years, just higher than that of the control group, with a mean age of 26.28 ± 4.31 years. Most of the study participants were less than 30 years old, with 65% among cases and 78% among controls. Most participants belonged to other backward castes in both groups, followed by scheduled castes. Nearly half of the cases (46%) and controls (54%) were educated up to the primary standard, and participants with a higher education qualification were 13% in cases and 5% in the control group. The education status of the husbands of the study participants had a similar distribution to that of the pregnant mothers, with the majority of them educated up to primary in both groups. At the same time, few were highly educated. 40% of the cases belonged to nuclear families, whereas 36% of controls belonged to joint families. The case group had more nuclear families as compared to the control group. The proportion of husbands working as laborers was higher in both cases and control groups. A large share of participants belonged to the lower middle and middle socioeconomic categories. The proportion of cases was higher in pregnant women < 30 years, with education up to the primary standard, belonging to the nuclear family, husbands educated up to the primary standard, working as laborers, and belonging to lower middle socioeconomic status (Table 1).

**Table 1:** Sociodemographic factors associated with pre-eclampsia

Factors	Cases (n=100)	Controls (n=100)	Chi-square value	p-value
Age (in Years)				
<30	65	78	4.147	0.042
≥30	35	22		
Caste				
Scheduled Tribe	15	8	2.534	0.469
Scheduled Caste	33	38		
Other Backward Caste	38	40		
General	14	14		
Education of mother				
Up to Primary	46	54	4.196	0.123
Secondary	41	41		
Higher	13	05		
Type of family				
Nuclear	40	35	1.481	0.477
Joint	28	36		
Extended	32	29		
Education of Husband				
Up to Primary	43	47	5.498	0.064
Secondary	35	43		
Higher	22	H10		
Occupation of husband				
Laborer	41	51	7.474	0.058
Govt/Private Employee	35	18		
Farmer	18	24		
Businessman	6	7		
Socioeconomic Status				
Upper	4	3	3.370	0.498
Upper Middle	6	9		
Middle	29	38		
Lower Middle	46	40		
Lower	15	10		

More pregnant women with a family history of hypertension (HTN) became pre-eclamptic, i.e., 28% among cases than 7% among controls. More number of mothers with a family history of diabetes mellitus (DM) developed pre-eclampsia (8%) than those without pre-eclampsia (1%). Most participants with a history of chronic hypertension became pre-eclamptic, i.e., 12% of cases and 1% of controls. The primigravida proportion in the study was higher among both cases and controls than multigravida. The proportion of participants with a history of previous abortion was more among controls. Both the groups had a higher number of women with blood group O, but those with the AB blood group were more among the cases (20%) than controls (8%). Seventy-six percent of the cases were anemic, and 66% among the controls (Table 2).

**Table 2:** Medical and obstetric factors associated with pre-eclampsia

Factors	Cases (n=100)	Controls (n=100)	Chi square value	p-value
Family History of Hypertension				
Yes	28	7	15.273	0.001
No	72	93		
Family History of Diabetes Mellitus				
Yes	8	1	---	0.035 (Fishers exact test)
No	92	99		
History of Hypertension				
Yes	12	1	9.955	0.002
No	88	99		
Gravida				
Primigravida	62	52	2.040	0.153
Multigravida	38	48		
H/O Abortion				
Yes	13	16	0.363	0.547
No	87	84		
Blood Group				
AB	20	8	10.237	0.017
A	12	21		
B	29	21		
O	39	50		
Anemia				
Yes	76	66	2.428	0.119
No	24	34		

Univariate and multivariable logistic regression analysis for the likelihood of pre-eclampsia

The variables with the p-value less than 0.1 during the Chi-square test were entered into univariate, multivariable logistic regression, and the risk factors were analyzed. On multivariable analysis, Pregnant women with a family history of hypertension had a 4.152 times higher risk of getting pre-eclampsia. A history of chronic hypertension (AOR = 13.737) was found to be independently associated with pre-eclampsia. Women with the AB blood group were found to be at 3.569 times higher risk than those with the O blood group of getting pre-eclampsia. The binary logistic regression model was significant with a chi-square of 49.055, a degree of freedom of 12, and a p-value of 0. 001. The model explained 29% of Nagelkerke R2 of the variance in pre-eclampsia and correctly classified 72.5% of the cases (Table 3).

**Table 3:** Multivariable logistic regression analysis for the likelihood of pre-eclampsia HTN: Hypertension; DM: diabetes mellitus

Factors	Unadjusted Odds Ratio (95% CI)	Adjusted Odds Ratio (95% CI)	p-value
Age (≥30 yrs)	1.909(1.020 - 3.573)	1.780(0.876 - 3.616)	0.111
Education of Husband			
Up to Primary	----		0.041
Secondary	0.890(0.484 - 1.635)	0.602(0.281 - 1.287)	0.191
Higher	2.405(1.023 - 5.650)	2.103(0.775 - 5.709)	0.145
Occupation of Husband			
Laborer	----		0.672
Govt/Private Employee	2.419(1.199 - 4.878)	1.583(0.648 - 3.869)	0.314
Farmer	0.933(0.447 - 1.949)	0.874(0.378 - 2.020)	0.752
Businessman	1.066(0.332 - 3.419)	1.073(0.249 - 4.628)	0.924
Presence of Family h/o HTN	5.167(2.135 - 12.500)	4.152(1.448 - 11.906)	0.008
Presence of Family h/o DM	8.609(1.056 -70.170)	2.624(0.241 - 28.583)	0.429
Presence of h/o HTN	13.500(1.720 -105.932)	13.737(1.610-117.25)	0.017
Blood Group			
O	----		0.020
AB	3.205(1.276 - 8.048)	3.569(1.306 - 9.753)	0.013
A	0.733(0.321 - 1.669)	0.639(0.244 - 1.672)	0.361
B	1.770(0.879 - 3.567)	1.759(0.798 - 3.876)	0.161

## Discussion

In the present study, the presence of a family history of hypertension (AOR = 4.2), history of chronic hypertension (AOR = 13.7), and AB blood group (AOR = 3.6) were found to be significant risk factors for pre-eclampsia. The age group of ≥30 years was found to have a significant association with pre-eclampsia with COR 1.909, but on multivariable logistic regression with AOR 1.780, the age group was not found as an independent risk factor of pre-eclampsia. The results were similar to the hospital-based studies conducted in India by Bej et al. [12] and Ganesh et al. [8]. They also observed no significant association between maternal age and pre-eclampsia.

A family history of hypertension was found to be an independent risk factor for pre-eclampsia. Women with a family history of hypertension were at 4.15 times more risk of developing pre-eclampsia than those without any family history. Similar results were found in studies conducted by Ganesh et al. [8] showing an AOR of 5.48, Kurniawati et al. [13] with an AOR of 3.37, and Machano et al. [14] with an AOR of 6.45. These findings further strengthened our results of a nearly five times higher risk of pre-eclampsia in women with a family history of hypertension. Studies conducted by Goyal et al. [5] and Ramesh et al. [15] showed eight times and 16 times higher risk with a family history of hypertension. The higher risk was compared to the present study because the adjusted OR was not calculated in the above studies with further logistic regression analysis. The above findings were in line with the fact that a family history of hypertension reflects a genetic factor predisposition towards an increased risk of pre-eclampsia.

As per the present study, a history of chronic hypertension increases the risk of pre-eclampsia by 13.37%, equivalent to a study by Das et al. [9] in Nepal that showed an AOR of 13.64. Similar associations were found in several other Indian studies showing a 6-fold increase in pre-eclampsia risk with chronic hypertension [5,8]. The increased risk of pre-eclampsia with chronic hypertension might result from increased vascular resistance in hypertension, thereby causing endothelial cell dysfunction and leading to pre-eclampsia.

In the present study, pregnant women with the AB blood group were at a higher risk of about 3.5 times compared to those with the O blood group. Manjunatha et al. [16] observed similar results in a study, "The relationship between maternal blood group and pre-eclampsia" [16]. A systematic review and meta-analysis by Li et al. to find the association between the ABO blood group and pre-eclampsia also suggested that pregnant women with the AB blood group were more likely to develop pre-eclampsia [17]. The AB blood group is associated with an increased risk of thrombotic events, which might cause increased risk of pregnancy-induced hypertension. 

Our study has limitations in the fact that it is a hospital-based case-control study conducted in a tertiary care center where the cases are likely to be of severe form as they are referred from other hospitals. 

## Conclusions

Risk factors identified in the present study can be used during routine antenatal check-ups to identify women at risk of pre-eclampsia early. Giving more focus to at-risk mothers and providing them with better antenatal care can help minimize the complications of pre-eclampsia in both mother and fetus and also help in preventing maternal deaths due to hypertensive disorders of pregnancy.
